# Westward Spread of *Echinococcus multilocularis* in Foxes, France, 2005–2010

**DOI:** 10.3201/eid1812.120219

**Published:** 2012-12

**Authors:** Benoît Combes, Sébastien Comte, Vincent Raton, Francis Raoul, Franck Boué, Gérald Umhang, Stéphanie Favier, Charlotte Dunoyer, Natacha Woronoff, Patrick Giraudoux

**Affiliations:** Author affiliations: Entente for the Control of Zoonoses, Nancy, France (B. Combes, S. Comte, V. Raton, S. Favier);; University of Franche-Comté/National Center for Scientific Research, Besançon, France (F. Raoul, P. Giraudoux);; Institut Universitaire de France, Paris (P. Giraudoux);; French Agency for Food, Environmental and Occupational Health and Safety, Nancy (F. Boué, G. Umhang);; National Game Federation, Issy-les-Moulineaux, France (C. Dunoyer);; Departmental Veterinary Laboratories Manager Association, Besançon (N. Woronoff)

**Keywords:** Cestode parasite, human alveolar echinococcosis, Vulpes, Echinococcus multilocularis transmission, prevalence, disease, fox, France, Europe, parasites, zoonoses

## Abstract

During 2005–2010, we investigated *Echinococcus multilocularis* infection within fox populations in a large area in France. The parasite is much more widely distributed than hitherto thought, spreading west, with a much higher prevalence than previously reported. The parasite also is present in the large conurbation of Paris.

*Echinococcus multilocularis* is the causative agent of the parasitic zoonosis alveolar echinococcosis. The adult stage of this cestode is found mostly in the digestive tract of the red fox (*Vulpes vulpes*) (*1*). Parasite eggs, expelled in feces, are the only external living stage of the parasite life cycle. Once ingested by small mammals, they migrate to the liver and proliferate, forming protoscolices in multivesicular cysts. The life cycle is completed when a definitive host (usually canid) preys on an infected intermediate host (mostly rodent). Epidemiologic studies indicate that humans can be infected by eating raw vegetables contaminated by infected fox or dog feces or by direct contact with an infected fox or dog (*2*). Despite the low incidence of human alveolar echinococcosis in Europe (0.02–0.18 cases/100,000 inhabitants [*3*]) the zoonotic potential of the fox tapeworm, in terms of persistence and pathogenicity, poses a major parasitic threat to human health in nontropical regions (*4*).

Three main trends have been reported in the past decade in Europe. First, *E. multilocularis* prevalence has increased in foxes within areas to which it is known to be endemic (*5*), seemingly linked with the increase of fox population densities in Germany and Switzerland (*6*). Second, the geographic distribution of *E. multilocularis* in foxes has extended toward southern, northern, and eastern countries where it had not previously been detected; the most recent are northern Italy (*7*); Svalbard, Norway (*8*); and Sweden in 2011 (*9*). Third, the geographic distribution of echinococcosis has extended toward Russia and neighboring countries (*10*), including the Baltic states.

Until now, the eastern part of the French territory was considered the western limit of the European echinococcosis-endemic area. At the end of the 1990s, *E. multilocularis* in foxes was reported in only 10 of the 95 French departments (Figure 1). Studies conducted in the neighboring departments (departments 08, 21, 38, 52, 69, and 74) by sedimentation and counting technique (*11*) did not detect infection in foxes. However, since 1997, new cases of human echinococcosis have been recorded in areas without known infection of local fox populations (departments 01, 03, 07, 08, 12, 21, 23, 31, 35, 61, 44, 59, 61, 76, and 95) (*2*).

**Figure 1:**
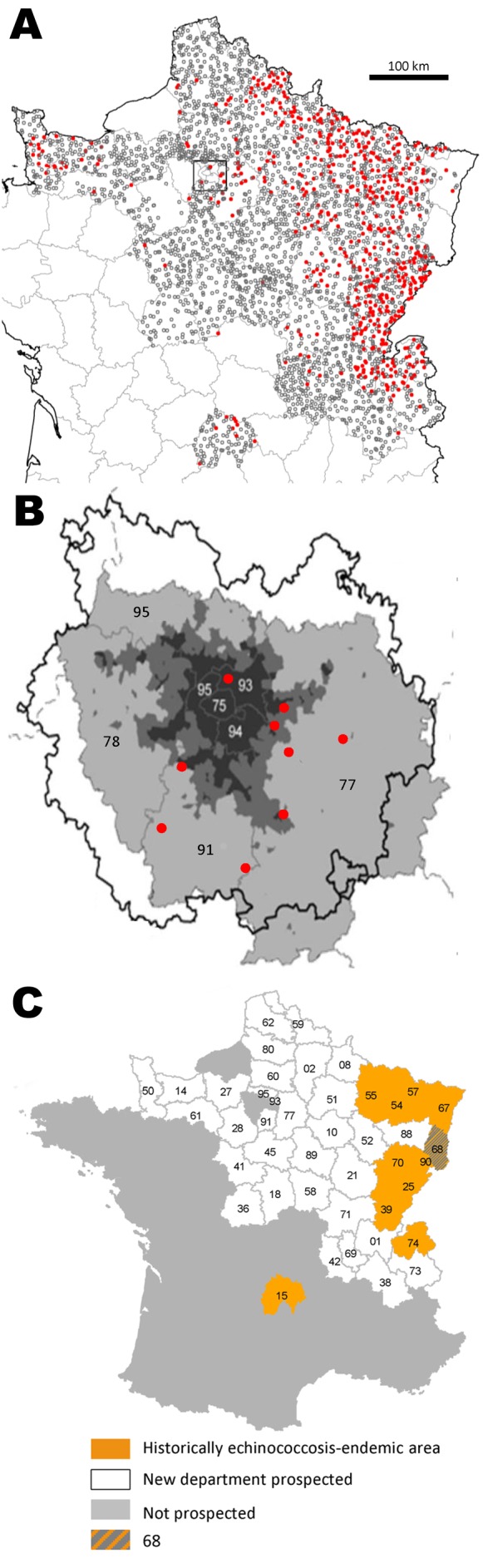
Fox locations (A, B) and department map (C), France, 2005–2010. Numbers in B and C are department national identification numbers. Panel B shows a close-up view of the departments of the Paris conurbation. Red circle, *Echinococcosus multilocularis*–positive fox; white circle, *E. multilocularis*–negative fox; dark gray, area totally urbanized (75 is Paris intra muros); medium gray, area intensively urbanized; light gray, periurban landscapes. C) Department 68 belongs to the historically echinococcosis-endemic area but could not be explored for the current study.

We present the results of a large-scale survey of *E. multilocularis* infection in foxes in France. Our study was conducted in 42 departments covering an area of 239,178 km^2^ representing almost all of northeastern France.

## The Study

During 2005–2010 (time span needed to cover the study area) and during the months more favorable for infection (October–April [*3*]), foxes were either shot at night or trapped. The sampling size was chosen to collect ≈100 foxes from each department. Therefore, a grid of 5 km × 5 km to 10 km × 10 km, depending on the department size, was superimposed over the sampling area, and no more than 1 fox was collected in each square. The geographic district where the sample was taken was then noted, and each fox was randomly allocated geographic coordinates within the commune (a French administrative division of 10–100 km^2^).

Adult *E. multilocularis* worms were identified in departmental veterinary laboratories. Staff were trained by the Anses-Nancy laboratory (National Reference Laboratory for echinococcoses); that laboratory also confirmed any unrecognized specimens. For time- and cost-effectiveness during the analysis, we used the segmental and sedimentation counting technique (*12*).

We used the χ^2^ test to compare *E. multilocularis* prevalence between departments. The distribution of *E. multilocularis* prevalence in foxes was modeled against geographic coordinates by using a generalized additive model with a logistic link function and a thin plate regression spline on 300 knots (*13*). Analyses and graphic displays were conducted by using ArcGIS 9.3, R 2.14.0 and the R packages maptools 0.8–10, mgcv 1.7–12, sp. 0.9–91, and splancs 2.01–29.

A total of 3,307 foxes were collected (Table 1). Eighty-five could not be assigned a commune code and were not kept for further analysis, except to compute *E. multilocularis* prevalence in departments. The mean number of foxes collected by department was 84.95 (± SD 25.76), which represents a mean of 1.56 foxes per 100 km^2^ (± SD 0.57). For 4 departments, (36, 61, 67, and 69), full sampling could not be completed because of technical and/or administrative reasons. Urban areas, such as departments 93, 95, and 91, also were undersampled because of human population density and high urbanization, all factors preventing easy fox sampling.

**Table 1:** Fox prevalence by department, France, 2005–2010

Department no., name	Total no. foxes	Prevalence, % (95% CI)	Density of collected foxes, no./100 km^2^
01-Ain	98	20 (13–30)	1.7
02-Aisne	89	20 (13–30)	1.22
08-Ardennes	91	36 (27–47)	1.85
10-Aube	99	12 (7–21)	1.68
14-Calvados	96	14 (8–22)	1.73
15-Cantal*	97	9 (5–17)	1.68
18-Cher	74	1 (0–8)	1.55
21-Cote d'Or	72	21 (12–32)	0.85
25-Doubs*	113	53 (44–62)	2.21
27-Eure	93	0 (0–5)	1.66
28-Eure et Loire	42	0 (0–10)	0.97
36-Indre	52	0 (0–9)	1.03
38-Isere	89	1 (0–7)	1.2
39-Jura*	102	52 (42–62)	2.02
41-Loire et Cher	86	2 (0–9)	1.47
42-Loire	97	1 (0–6)	2.06
45-Loiret	100	0 (0–5)	1.53
50-Manche	81	15 (8–25)	1.35
51-Marne	103	19 (13–29)	1.26
52-Haute Marne	94	14 (8–23)	1.51
54-Meurthe et Moselle*	84	54 (42–64)	1.8
55-Meuse*	104	41 (32–51)	1.67
57-Moselle*	103	34 (25–44)	1.65
58-Nievre	110	1 (0–6)	1.74
59-Nord	96	20 (13–29)	1.74
60-Oise	87	7 (3–15)	1.53
61-Orne	55	4 (1–14)	0.93
62-Pas de Calais	90	0 (0–5)	1.34
67-Bas Rhin*	7	29 (5–70)	0.44
69-Rhone	48	8 (3–21)	1.69
70-Haute Saone*	81	36 (26–47)	1.54
71-Saone et Loire	79	9 (4–18)	1.13
73-Savoie	75	11 (5–20)	1.26
74-Haute Savoie*	73	49 (38–61)	1.76
77-Seine et Marne	55	29 (18–43)	1
80-Somme	89	8 (3–16)	1.68
88-Vosges	90	24 (16–35)	1.7
89-Yonne	97	0 (0–5)	1.75
90-Territoire de Belfort*	25	32 (16–54)	4.09
91-Essonne†	41	7 (2–21)	2.37
93-Seine Saint Denis†	6	17 (1–64)	2.53
95-Val d'Oise†	44	0 (0–10)	3.59
Historical endemic area	789	41 (35–41)	1.56
Total	3307	17 (16–19)	1.38

*Department belonging to the historically echinococcosis-endemic area. †Department of the Paris capital conurbation (Figure 1).

**Figure 2:**
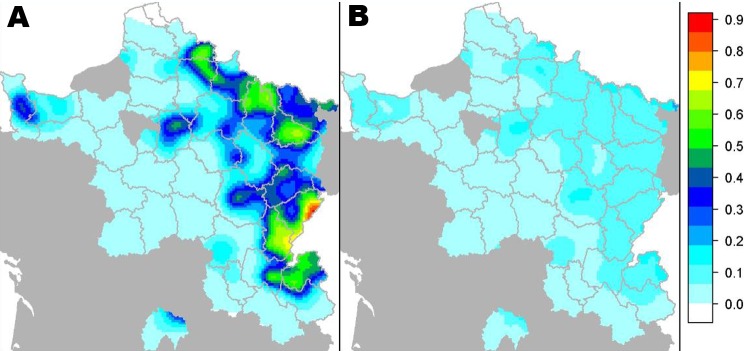
Model-predicted prevalence (A) and standard error (B) of *Echinococcus multilocularis* in foxes, France, 2005–2010. 1 = 100%.

We confirmed *E. multilocularis* in foxes in 35 departments (Figure 2). The prevalence varied widely among departments, from 0 (95% CI 0–5%) to 54% (95% CI 42%–64%) (Table 1) but was locally higher in some areas (Figure 2). The mean prevalence in the entire studied area was 17% (n = 3,307; 95% CI 16%–19%). The prevalence in the historically echinococcosis-endemic area was 41% (n = 789; 95% CI 37%–44%) and represented >55% of all infected foxes and <21% of the total area studied. Furthermore, in comparing our results with those of earlier similar studies during the same season with the same technique, we detected a significant increase of *E. multilocularis* prevalence in foxes over time in most of these departments (Table 2).

**Table 2:** Changes in fox prevalence over time, France

Department	Total no. foxes collected		Prevalence, %		p value*
	1984–1987	2006–2010	1984–1987	2006–2010	
54/57	153	187	28	43	0.05
39	146	102	18	52	0.0002
25	37	116	46	52	0.85
67	192	21	4	19	0.04

*p value of the χ^2^ comparing the 2 periods.

## Conclusions

Our study confirms the presence of *E. multilocularis* in areas where it is known to be endemic and indicates its presence in 25 additional departments. However, we cannot discard the possibility that *E. multilocularis* was present but remained undetected during the 1980s–1990s. That *E. multilocularis* could have remained undetected if it were not already at a very low prevalence in general is doubtful. Isolated human cases recorded in the early 2000s outside areas to which it is known to be endemic corroborate this possibility (*3*). The same uncertainty applies in other parts of Europe (*14*). Taken as a whole, these findings indicate that the transmission intensity of *E. multilocularis* through fox populations in the occidental part of the European focus area is likely to have increased during the late 1990s and led to a much higher average prevalence than previously reported. Furthermore, infected foxes close to large-scale conurbations, such as Paris and its large suburban surrounding departments (93, 91, and 77) (Figure 1) amounting to 11,728,240 inhabitants, may create new conditions for human exposure similar to those already described in other highly urbanized cities, such as in Switzerland, Germany, and eastern France (Nancy), but on a much larger scale.

We believe that the public needs to be proactively informed and protected, including through awareness initiatives among urban residents and, in specific areas (*15*), more direct action toward the parasite may be considered. Monitoring the possible further extension of the parasite westward and southward and the evolution of prevalence in foxes in the historically and the newly echinococcosis-endemic areas also are essential.
